# Supplementary material for: Content-rich biological network constructed by mining PubMed abstracts
Source: BMC Bioinformatics. 2004 Oct 8;5:147. doi: 10.1186/1471-2105-5-147 (PMC528731; doi:10.1186/1471-2105-5-147)
Supplement: Additional File 5 — The original Chilibot query results of the term "long-term potentiation (LTP)" and 22 other terms, limiting the latest references analyzed to the years 1990, 1995, 2000, and 2004. [file 1471-2105-5-147-S5.bz2 › chilibotAdditionalFile5/ltp2000/html/TRKA.html]

 


**TRKA** (Input: TRKA ) 

---


**Google Searches:** Entire Web  | EDU domain only  | PDF files only

.

**External Links:** OMIM |
LocusLink
| Swissprot
| GeneCards

  
**Maps of TRKA**

Simple
Complete
graph in
radiant
tree
square
layout.

**New Hypothesis !**

**Synonyms** 

- trk a   [PubMed]

- trka   [PubMed]

**Synopsis**

- We have found that **TRKA** is the gene responsible for CIPA.  Hum Genet, 2000 
  [26]

- These results suggest that the NGF effect is mediated by the high affinity NGF receptor, Trk A  [**TRKA**] .  J Biol Chem, 1997 
  [23]

- Hepatocyte RNA was found to contain BDNF, NT 3, NT 4 5 mRNA which is compatible with the immunohistochemical findings and Trk A  [**TRKA**]  mRNA.  Hepatology, 2001 
  [22]

- mRNA for **TrkA**, TrkB and p75 receptors was expressed in the injured nerve, suggesting that these specific receptors can mediate the effects of the T cell derived NTs.  J Autoimmun, 2000 
  [22]

- These observations suggest that **TrkA** can activate different survival signalling pathways, which can interfere with specific apoptotic pathways.  Oncogene, 1998 
  [19]

- Results showed that **trkA**, full length trkB, and trkC receptors were NOT present in the lesion site but had a normal expression pattern in uninjured parts of the spinal cord.  Exp Neurol, 2000 
  [16]

- Mechanistically, NGF stimulation of PC12 cells resulted in a weak or possibly indirect association between **trkA** and PI 3 kinase.  J Neurosci Res, 1995 
  [14]

- Furthermore, even after transfection with exogenous TRK A  [**TRKA**] , early responses were restored but later events such as neurite outgrowth did NOT occur, suggesting that downstream responsiveness is blocked as well.  Prog Clin Biol Res, 1994 
  [12]

- These results indicate that in developing cortical neurons, activation of AMPA KA receptors by KA may induce expression, followed by activation of **TrkA** via PLC signaling and intracellular calcium elevation and hence increase reception of NGF on KA challenged neurons.  J Neurochem, 2000 
  [11]

- Furthermore, our data directly reveal distinct patterns of trk receptor expression by individual sensory neurons from the time of their inception with all early arising cells initially being trkC, some subsets of whom coexpress either **trkA** or trkB or both.  Dev Biol, 2000 
  [11]

- Furthermore, TrkC14 and TrkC25 have only a low intrinsic tyrosine kinase activity, and insertion of the TrkC14 kinase insert into **TrkA** at an equivalent position results in a dramatic reduction of the kinase activity and signaling capabilities of **TrkA**.  J Biol Chem, 1996 
  [11]

- Uniform exposure of **TrkA** expressing neurons to NGF resulted in a cross desensitization of turning responses induced by a gradient of netrin 1, brain derived neurotrophic factor BDNF, or myelin associated glycoprotein MAG  Neuron, 1999 
  [11]

- These results clearly implicate c Crk II in the NGF signaling pathway and support the concept that more than one signaling molecule known to participate in the activation of Ras associates with **TrkA** upon NGF treatment.  Oncogene, 1996 
  [11]

- NGF does NOT result in **TrkA** phosphorylation.  Brain Res Dev Brain Res, 1996 
  [11]

- We find that **TrkA**, TrkC and p75 mRNAs are restricted to the cell soma but in addition, p75 mRNA labelling extends in average for 8 microm within the proximal dendrites of 34% of the labelled neurons.  Neuroreport, 2000 
  [11]
